# Sex Ratio Estimations of Loggerhead Sea Turtle Hatchlings at Kuriat Islands, Tunisia: Can Minor Nesting Sites Contribute to Compensate Globally Female-Biased Sex Ratio?

**DOI:** 10.1155/2014/419410

**Published:** 2014-10-14

**Authors:** Imed Jribi, Mohamed Nejmeddine Bradai

**Affiliations:** ^1^Sfax Faculty of Sciences, University of Sfax, P.O. Box 1171, 3000 Sfax, Tunisia; ^2^National Institute of Sea Sciences and Technologies, 3000 Sfax, Tunisia

## Abstract

Hatchling sex ratios in the loggerhead turtle *Caretta caretta* were estimated by placing electronic temperature recorders in seven nests at Kuriat islands (Tunisia) during the 2013 nesting season. Based on the mean temperatures during the middle third of the incubation period, and on incubation duration, the sex ratio of hatchlings at Kuriat islands was highly male-biased. Presently, the majority of hatchling sex ratio studies are focused on major nesting areas, whereby the sex ratios are universally believed to be heavily female-biased. Here we present findings from a minor nesting site in the Mediterranean, where the hatchling sex ratio was found to be male-biased, suggesting a potential difference between major and minor nesting sites.

## 1. Introduction

Evolutionary theory [1] suggests that male and female offspring should be produced in equal proportions (Fisherian equilibrium). There should be selective pressure for this to be the case, because if one sex became rarer, that sex would have proportionally more opportunities to reproduce and would therefore contribute a higher proportion of offspring to the gene pool. The benefit of being the rarer sex should continue until the sex ratio reaches 1 : 1, an “evolutionary stable strategy” [2]. “Fisherian” equilibrium however, includes the total parent's investment until the end of the parental care period. In sea turtles the parental care ends at the egg laying phase, which means that determination of sex ratio is largely controlled by environmental parameters including the nest temperature.

Sex determination is the initial event in which undifferentiated gonads opt for either ovarian or testicular differentiation. This process in most vertebrate species is genotypic (GSD for genotypic sex determination), leaving little scope for deviation from a balanced primary sex ratio [3].

Sea turtles, like many other members of the Class Reptilia, possess temperature-dependent sex determination, or TSD (reviewed by Mrosovsky [4]). Research has indicated that sexual differentiation in TSD species is determined by the temperature at which the eggs are incubated, the crucial period being the approximate middle third of development [5, 6]. The point at which a balanced sex ratio occurs is known as the pivotal temperature; more females result from temperatures above the pivotal temperature and more males from cooler temperatures (see [7] for a review).

TSD is an important factor to consider in the conservation of the sea turtle species. Not only does this aspect of their development affect the natural populations, but should be an important consideration when designing nest relocation and hatchery programs. It is imperative that an accurate and nonlethal sexing technique is developed so that sex ratios can be monitored in conservation programs [8].

The determination of sex and, hence, the sex ratio of hatchlings are very significant basic information in marine turtle population dynamics [3, 9, 10]. It should therefore be taken into account in any conservation planning of nesting beaches in order to conserve the “population's sexual structure” and act in an appropriate manner for the protection of these endangered reptiles, especially in the context of current global warming [11, 12]. Indeed, in species with TSD, the sex-determining pathway is extremely sensitive to temperature [13]. The transitional range of temperature within which the complement of offspring sex shifts from 100% male to 100% female (or vice versa) is generally less than 2°C and may be less than 1°C [14], while the mean warming predicted on a scale of 100 years is 2°C [15].

Investigations of the sex ratio of all three classes of loggerhead turtles (hatchlings, juveniles, and adults) have suggested prioritizing the study of the ecological effects of anthropogenic climate change on marine turtles [10]. Such studies have recently begun in the Mediterranean region, but the results cannot yet be considered conclusive at the regional population scale [9]. The loggerhead hatchling sex ratios are estimated to be female-biased on most beaches in the Mediterranean [16–20] in contrast with more balanced sex ratios in adult and juvenile [21].

Among the world's seven marine turtle species, three species are regularly observed in the Mediterranean: the loggerhead turtle,* Caretta caretta*, the green turtle,* Chelonia mydas,* and the leatherback turtle,* Dermochelys coriacea.*

All three marine turtle species mentioned were reported in Tunisian waters, but only the loggerhead turtle is a nesting species on some beaches [22–24]. Nesting activity in Tunisia was mentioned in the literature but not based on systematic surveys [25–27]. The nesting of the loggerhead turtle (*Caretta caretta*) was first recorded in 1988 on the beach situated between Ras Dimas and Mahdia and on the island Great Kuriat [28], the latter of which is considered as the most important nesting site in the country [23, 24]. Few nests were sometimes laid in other beaches along the Tunisian coasts but the nesting is not regularly observed.

Since 1997, the beaches of both Great and Small Kuriat islands have been monitored, to study nesting density and protect nests, nesting females and hatchlings and to determine reproductive parameters. Despite the importance of the Kuriat islands in Tunisia, their beaches should be considered as minor Mediterranean nesting sites for the loggerhead turtle.

Studies of hatchlings sex-ratio in the Mediterranean concerned mainly major nesting sites such as Zakynthos [3] and Kyparissia Bay [29, 30] in Greece, Alagadi in Cyprus [16], Fethiye [31], Patara beaches [17] in Turkey, Sirte in Libya [19] but few studies have been conducted for minor nesting sites such as Sicily in Italy [32]. The protection and the study of these minor nesting sites are informative because they can give an appreciable contribution to sea turtle biology, both in number and in genetic diversity [24]. Moreover, minor site studies may also reveal novel sex-ratio data for the Mediterranean suggesting a much less female-biased ratio than previously believed.

Recalling the articles of the SPA protocol and the revised action plan on marine turtles in the Mediterranean [33], taking into account the new developments concerning conservation measures based on scientific groundwork, and considering the potential effects of global warming on future population structure and on the dynamics of these endangered species [11, 12], the present study aimed to provide data on hatchling sex ratio estimation from beaches of Great and Small Kuriat, which are the most important nesting grounds for loggerhead turtles in Tunisia. Since the sex of marine turtle hatchlings cannot be assessed from external morphology as it needs direct observation of the gonads which imply to kill the individuals and since sacrificing hatchlings was not an option for ethical and conservation reasons, we used incubation duration of clutches and the mean temperatures during the middle third of the incubation period as indirect methods for predicting the sex ratios within the nests.

## 2. Material and Methods

The Tunisian coast spans approximately 1200 km, representing about 2.5% of the coasts of the Mediterranean (about 46000 km) [34]. In the north, most of coasts are rocky while in the centre and in the south, the coasts are mostly sandy with a very large continental shelf in the Gulf of Gabes.

**Figure 1:**
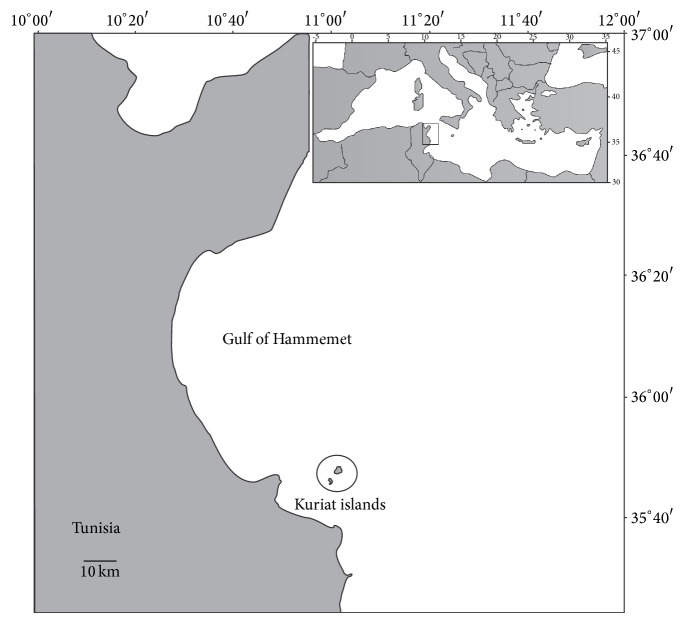
Geographic position of Kuriat islands.

The Kuriat islands are situated in the centre of Tunisia (Figure 1) (35° 48′ 05′′ N, 11° 02′ 05′′ E) and lie 18 km from the coast of Monastir. They consist of two small islands: the Small Kuriat (Kuria Sgira) which is* ca*. 0.7 km^2^ and the Great Kuriat (Kuria Kbira) which is* ca*. 2.7 km^2^ in area. Small Kuriat has a total of 1500 m of sandy beach situated in the north-eastern and east parts of the island whereas the rest of the coastline is rocky or marshy. Almost one-third of the Great Kuriat shoreline is rocky and large deposits of sea grass,* Posidonia oceanica*, and detritus further restrict the accessible nesting sites particularly in the south and the south-western beaches. The principal nesting beach lies on the western and southern coast and it is almost 3000 m in length.

The nesting season in Kuriat islands generally starts at the beginning of June and ends at the middle of August. Deposition of nests occurred in June and mainly in July, whereas nesting in August remains rare [24]. The fieldwork was conducted during the summer months of 2013 on the beaches of both Great and Small Kuriat. Nesting and hatching activity were observed over the beaches as part of the long-term monitoring undertaken by the Tunisian Sea Turtle Programme (TunSTP) since 1997 following a convention signed every year between the National Institute of Sea Sciences and Technologies (INSTM), the Regional Activity Centre for Specially Protected Areas (RAC/SPA), and the Agency of Protection and Management of the Littoral (APAL). The beach is surveyed throughout the nesting season, on both islands. A team of three to four persons (researchers, students, and volunteers) is permanently present during the season in order to record female laying or hatchlings emergence dates. Each nest was located by walking on the beach and the precise GPS position was recorded in order to locate the nest and identify its first hatchling emergence date. For the purpose of this study, incubation duration is defined as the period in days between observation of the newly laid nest and the first record of emergence, by either direct observation of hatchlings or their crawl tracks emerging from nests.

Temperatures in seven loggerhead turtle nests were examined using a Hobo data logger Pendant UA Temp/Alarm (Prosensor, Fr) from July to September 2013 on beaches of Small and Great Kuriat (when most clutches are in their thermosensitive stage for sex determination). In order to cover the entire nesting site so that the study could be representative of the area, the data loggers were distributed to the beaches according to the nesting densities of previous four seasons: 4 in Great Kuriat and 3 in Small Kuriat. Within each nesting beach, nests were selected so that the data loggers were homogeneously spread along the beach length. Furthermore, the locations of studied nests were also chosen within the area of mean distance from the wave line, based on data of nests laid at previous seasons. In all cases temperature data loggers were placed into the centre of the nests before the start of the second third of the incubation period either during the egg-laying, in the following morning or a few days after the discovery of the nest. In this latter case, to avoid disturbing the nest, a small hole was made adjacent to the egg chamber, without excavating the nest, 3-4 eggs were carefully removed in order to place the data logger and then they were returned to their initial positions, with the exact orientation. Temperatures at three levels (top, middle, and bottom) in two nests were also recorded.

In order to study the effect of metabolic heating, a second temperature data logger was buried adjacent to each nest (approximately 1 m from the nest at the same depth and the same distance from the sea). All loggers were programmed to record a reading every 15 minutes.

Nest contents were excavated within a specific period after the first hatchling emergence, as suggested by Adam et al. [35]; nest depths were measured and data loggers were retrieved. The total number of eggs (the number of eggs laid into the nest) and the hatching success were calculated by counting unhatched eggs, dead hatchlings in eggs, and dead hatchlings in nests. Empty eggshells (>50% complete) were characterized as successful hatching. The hatching success (%) is calculated as follows: (empty eggshells/total number of eggs) ∗ 100. The middle third of the incubation period was calculated on the basis of the incubation period mentioned above.

Two methods were used to estimate the sex ratio of hatched loggerhead turtles. The first used the mean temperature during the middle third of the incubation period, while the second used the incubation duration. The curves used for estimation of sex ratio as functions of incubation duration and temperature during the second third of the incubation duration were those of Mrosovsky et al. [29] adapted to the field. This choice is based on the fact that turtles from Greece and those of Tunisia are part of the same Mediterranean population and have the same geographic range. It is also based on the fact that pivotal temperature in marine turtles is a relatively conservative characteristic [13, 30]. The sex ratio curve (% of females) as a function of the mean temperature during the second third of incubation duration was adapted to the field by adding 0.4°C [29], which corresponds to the difference between ambient temperature and egg temperature. The sex ratio curve (% of females) as a function of incubation duration [29] was also adapted to the field by adding 4 days, which corresponds to the interval between hatching and the emergence of hatchlings at the sand surface [36]. The equations of the two curves used (after corrections) calculated by Jribi et al. [19] were used and the exact values of sex ratios were derived.

These equations are writen as follows.

The equation of sex ratio as a function of temperature derived from Mrosovsky et al. [29] is written as follows:

(1) $$\begin{array}{c} Y=\frac{100.06}{1+\text{Exp}\left(+188.78-6.37*X\right)}, \end{array}$$

where *Y* is the sex ratio and *X* is the temperature.

The equation of sex ratio as a function of incubation duration derived also from Mrosovsky et al. [29] is written as follows:

(2) $$\begin{array}{c} Y=\frac{99.88}{1+\text{Exp}\left(-103.34+1.82*X\right)}, \end{array}$$

where *Y* is the sex ratio and *X* is the incubation duration.

## 3. Results

During the 2013 nesting season, 22 nests were recorded in Kuriat islands, 13 in Great Kuriat and 9 in Small Kuriat. This number exceeds slightly the average registered since the start of monitoring in 1997 (average = 16.8; SD = 8.8; *N* = 17). All these nests were controlled until their emergence and excavation.  Table 1 presents the information on the seven studied nests.

Emergence success was null in two studied nests. In nest GK7, the majority of eggs were unhatched (90% of total) and in nest SK5, almost all embryos were dead at a late stage of embryonic development for unknown reason.

The information on temperature recorded in the studied nests and in adjacent sand is presented in Table 2. Two temperature data loggers buried in the sand were lost, their data are lacking in the table.

The mean temperature of the whole incubation period (each temperature data point recorded was used) for the 7 nests ranged from 27.46°C to 29.68°C. The maximum temperature increase during the incubation period was 6°C (for nest SK6, minimum of 24.7°C and maximum of 30.7°C).

The mean temperature in nests during the middle third of the incubation ranged from 27.6°C (Nest GK7) to 29.3°C (Nest GK4). The maximum temperature increase during this period was 2.29°C (nest SK6: minimum of 28.16°C and maximum of 30.46°C). The minimum temperature increase was 0.99°C (nest SK4: minimum of 27.96°C and maximum of 28.95°C) ignoring the nest GK7 (increase was 0.6°C) where the majority of eggs were unhatched.

**Figure 2:**
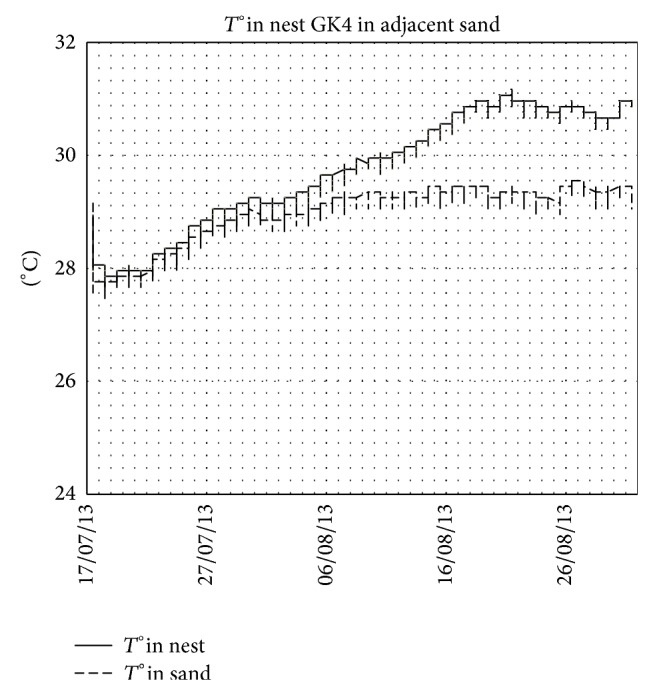
Temperatures in nest GK4 (normal nest) and in adjacent sand. Note the difference between inside and outside the nest due to metabolic heating.

**Figure 3:**
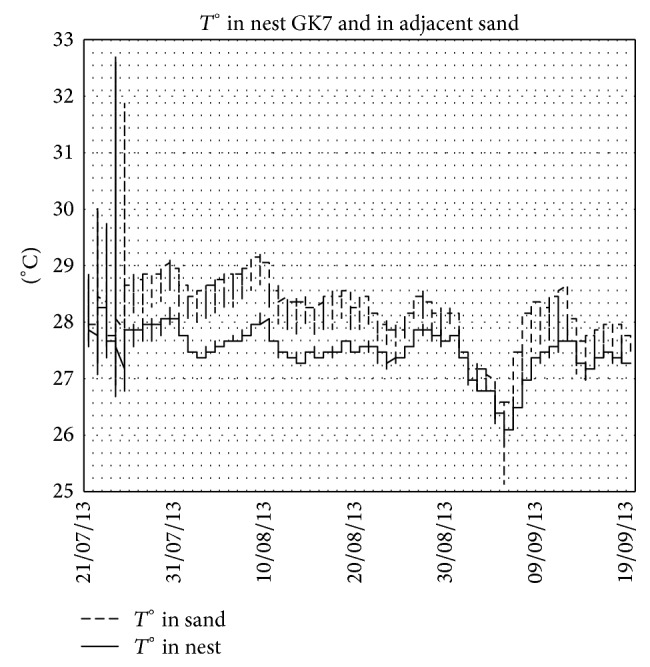
Temperatures in nest GK7 (with a majority of unhatched eggs) and in adjacent sand. Note the nest temperature is lower than outside temperature, lacking metabolic heating.

**Figure 4:**
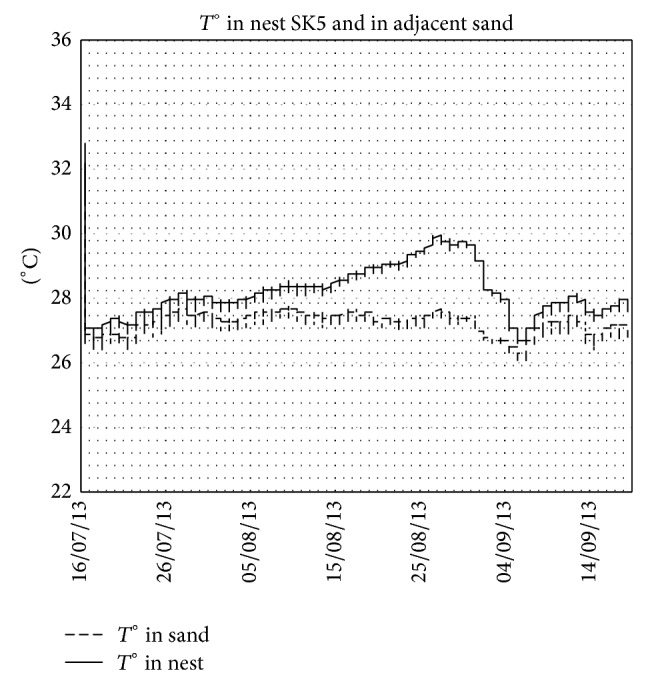
Temperatures in nest SK5 (majority of eggs with embryos died in late phase of development) and in adjacent sand. Note the lower difference between inside and outside of the nest after embryos died on 04/09/2013.

The mean temperature during the incubation period increased in the middle third of the incubation period compared with the first third and continued to increase during the last third. This is not the case for the adjacent sand (control), where air temperature controls the increase and decrease in soil temperature. This evolution of the temperature is observed in the case of nests in normal conditions (Figure 2 in nest GK4, e.g.). When the conditions are not normal, the patterns become different.  Figure 3 shows the temperatures in nest GK7 where the majority of eggs were unhatched and in adjacent sand and the Figure 4 shows the case of nest SK5 where the majority of embryos died in a late phase of development and in adjacent sand.

The study of temperature at different levels of a nest indicates, as expected, that temperatures decreased with increasing depth (Table 3). This parameter was studied in two nests (GK6 and GK8). In these two nests, no difference was recorded between temperature in the middle part of the nest and the mean temperature of the three levels of the nest (*t*-test, *t* = 2.76, *P* value = 0.125 for nest GK6 and *t* = −1.08, *P* value = 0.375 for nest GK8).

Recording temperature in both the sand and the nests allowed us to compare the temperatures at the same depth in both settings. During the total incubation duration, the daily mean sand temperatures were 0.33°C to 0.99°C lower than in loggerhead nests at the same time and depth. During the middle third of the incubation period, when sex is thought to be determined, the mean temperature difference between nest and sand was 0.56°C (*n* = 4; 0.21–1.44°C). In nests GK7 with majority of unhatched eggs, the difference was −0.53°C.

The estimated sex ratios of hatchlings for all studied nests from equations are shown in Table 4.

The sex ratio ranged between 0% (with the two methods) and 10% (with the *T*° method) or 40% (with the ID method).

All nests were predicted to produce more males. With ID methods, only one nest can produce females (40%). With *T*° method, only one nest can produce females (10%) with three others with very low proportion (1 or 2%). Comparison of the two methods indicates that sex ratios are not significantly different (*t* = −0.8765, *P* value = 0.43026). Comparison of the two methods for Great Kuriat and Small Kuriat taken separately also shows that there is no significant difference; *P* values of the *t*-test for the two beaches were, respectively, 0.46 and 0.5.

The analysis of the sex ratios estimated by the two methods shows that there was no difference between the two beaches of Great Kuriat and Small Kuriat (Kruskal-Wallis test, *H* = 0.125, *P* value = 0.711 for the *T*° method and *H* = 0.333, *P* value = 0.414 for the ID method).

Furthermore, the analysis of the results of the sex ratio for the nests laid in early July (1–15 July) and those laid in late July (16–31 July) showed no significant differences (Kruskal-Wallis test, *H* = 0.2813, *P* value = 0.5784 for the *T*° method and *H* = 0.75, *P* value = 0.2207 for the ID method).

## 4. Discussion

Nest temperature and incubation duration are the two approaches used for estimating the sex ratios of hatchlings born in the minor nesting site of Kuriat islands, Tunisia, during the 2013 nesting season. Although the results are comparable and close, the temperature method was more accurate, because the incubation duration method was based on the relationship between incubation duration and the temperature during the entire development period. Therefore, it is less accurate, as it is indirect and based not only on the middle third of the incubation duration when sex is thought to be determined. The other thirds may confound results in case of within clutch heterogeneous temperature regimes [19].

Temperatures from pilot experiments in two nests were measured at different parts (top, middle, and bottom). Results allowed us to claim that the mean temperature in the central part of the nest is representative of the whole nest sections. Recording only central parts of the nests therefore yielded the best estimation of sex ratios [8, 37] and allowed us to save more data loggers to be used at other nests.

During our study, we had the opportunity to monitor the temperature in nests at different states: (i) nests in normal conditions (Figure 2), (ii) nest with majority of unhatched eggs (Figure 3), and (iii) nest with majority of dead embryos at late stage of development (Figure 4). These three figures illustrate well the metabolic heating in the nest. Clutch temperatures closely followed the course of sand temperatures during the first third of incubation. The increasing discrepancy between nest and sand temperature afterwards is attributed to metabolic heating. In the absence of live embryos, there is no metabolic heating. During the middle third of incubation duration, this increase is estimated to be 0.56°C. Knowing that we measured the nest temperature at the centre of the clutch and that it has been shown, however, that temperature in loggerhead clutches is not evenly distributed [8, 37–41] and that the amount of metabolic heating is higher in the centre than at the sides of the clutch [39, 40], our results are likely to have overestimated the amount of metabolic heating experienced by the average egg. This potential bias is not likely to affect the sex ratio in Kuriat islands, as this heat increase is negligible [18].

The results of our study indicate that the primary sex ratio of hatchlings in 2013 was strongly male-biased in Kuriat islands (Tunisia). These results are in agreement with those of Casale et al. [32] in Sicily (a minor nesting site in Italy) and differ from the general pattern of producing female dominated sex ration in loggerhead marine turtle of the Mediterranean region and globally [7, 8, 12, 16, 17, 19, 30, 31, 37, 38]. These results confirm also the importance of studying minor nesting sites, because they can give an appreciable contribution, to male hatchlings number (sex ratio) and genetic diversity [24]. At first glance and taking into account the small area of minor nesting sites in the Mediterranean, like Kuriat islands, it can be assumed that they have no significant effect on the Mediterranean female-biased hatchlings production, but knowing that majority of published studies concentrated only on major nesting sites that attract more attention for protection effort, and small number of studies investigated this phenomenon in smaller nesting sites and the fact that both sexes of juvenile and adult in Mediterranean foraging grounds in the open sea have approximately equal proportions: 50% : 50% [21], results from minor nesting sites can be important and can give some elements of answer to the difference recorded in sex ratio among the different life stages of* Caretta caretta* in the Mediterranean which provoke their study more.

Taking into account that only females come to beach to lay and show a certain fidelity to their natal nesting sites and that major nesting sites are producing mainly females, we can infer that major nesting sites remain usually major and minor nesting sites with male-biased sex ratios remaining usually minor.

It would be then very important to continue the estimation of sex ratio in Kuriat islands to see if 2013 nesting season was exceptional or if the male-biased sex ratio is a character of the site. It is also very important to extend the estimations of sex ratio in order to cover the minor nesting sites, even if scattered over long coastal tracts, because they may contribute to a better understanding of the sex ratio patterns and may also represent important areas in future scenarios of climate change [11].

## Figures and Tables

**Table 1:** Information on studied nests.

Nest	Laying date	Start of monitoring	Cluch size	Emergence success	Incubation duration (days)
GK4	06-07	17-07	83	50.6	57
GK6	18-07	18-07	121	60.3	63
GK7	25-07	25-07	57	0.0	57∗
GK8	31-07	31-07	75	86.7	60
SK4	15-07	16-07	56	78.6	61
SK5	15-07	16-07	104	0.0	66∗
SK6	19-07	21-07	98	95.9	65

GK: Great Kuriat. SK: Small Kuriat. ∗Day of excavating the nest.

**Table 2:** Mean temperature in study nests and adjacent sand during different incubation periods. IP, incubation period. The data loggers in sand adjacent to nests 6 and 8 in Great Kuriat were lost.

Nest	In nest				In sand			
	Total IP	First third IP	Middle third IP	Last third IP	Total IP	First third IP	Middle third IP	Last third IP
GK4	29.68	27.97	29.29	30.68	28.90	27.84	28.92	29.25
GK6	29.03	28.19	28.98	29.88	—	—	—	—
GK7	27.46	27.76	27.55	27.10	27.99	28.55	28.08	27.37
GK8	28.24	28.60	28.04	28.08	—	—	—	—
SK4	28.49	27.68	28.59	29.08	28.16	28.10	28.38	28.00
SK5	28.13	27.62	28.82	27.99	27.14	27.13	27.38	26.91
SK6	28.78	28.42	28.89	28.97	28.37	28.70	28.67	27.79

**Table 3:** Mean temperature (°C) in different parts of the nests GK6 and GK8 during the incubation period. T, top; M, middle; B, bottom; and TSP, thermosensitive period.

Period	Mean temperature		
		Nest GK6	Nest GK8
Incubation period	T	29.19	28.64
	M	29.03	28.24
	B	28.19	27.96

Before TSP	T	28.87	29.25
	M	28.19	28.60
	B	27.44	28.33

TSP	T	29.2	28.45
	M	28.98	28.04
	B	28.1	27.81

After TSP	T	29.49	28.23
	M	29.88	28.08
	B	29.03	27.76

**Table 4:** Information on studied nests with estimated sex ratio (%♀).

Nest	Clush size	Emergence success	ID	*T*° (middle third ID)	Sex ratio (%♀) from *T*°	Sex ratio (%♀) from ID
GK4	83	50.6	57	29.29	10	40
GK6	121	60.3	63	28.98	2	0
GK7	57	0.0	57	27.55	0	—
GK8	75	86.7	60	28.04	0	0
SK4	56	78.6	61	28.59	0	0
SK5	104	0.0	66	28.82	1	—
SK6	98	95.9	65	28.89	1	0
Mean	84.9	53.2	61.3	28.6	2.0	8.0

ID: incubation duration.
